# Therapeutic plasma exchange in children with acute liver failure (ALF): is it time for incorporation into the ALF armamentarium?

**DOI:** 10.1007/s00467-021-05289-0

**Published:** 2021-10-14

**Authors:** Emma C. Alexander, Akash Deep

**Affiliations:** 1 Paediatric Intensive Care Unit, King’s College Hospital NHS Foundation Trust, Denmark Hill, London, UK; 2 Department of Women and Children’s Health, School of Life Course Sciences, King’s College London, London, UK

**Keywords:** Plasma exchange, Extracorporeal liver support, Acute liver failure, Paediatric intensive care unit, Children

## Abstract

Paediatric acute liver failure (PALF) is a rare but devastating condition with high mortality. An exaggerated inflammatory response is now recognised as pivotal in the pathogenesis and prognosis of ALF, with cytokine spill from the liver to systemic circulation implicated in development of multi-organ failure associated with ALF. With advances in medical management, especially critical care, there is an increasing trend towards spontaneous liver regeneration, averting the need for emergency liver transplantation or providing stability to the patient awaiting a graft. Hence, research is ongoing for therapies, including extracorporeal liver support devices, that can bridge patients to transplant or spontaneous liver recovery. Considering the immune-related pathogenesis and inflammatory phenotype of ALF, plasma exchange serves as an ideal liver assist device as it performs both the excretory and synthetic functions of the liver and, in addition, works as an immunomodulatory therapy by suppressing the early innate immune response in ALF. After a recent randomised controlled trial in adults demonstrated a beneficial effect of high-volume plasma exchange on clinical outcomes, this therapy was incorporated in European Association for the Study of Liver (EASL) recommendations for managing adult patients with ALF, but no guidelines exist for PALF. In this review, we discuss rationale, timing, practicalities, and existing evidence regarding the use of plasma exchange as an immunomodulatory treatment in PALF. We discuss controversies in delivery of this therapy as an extracorporeal device, and practicalities of use of plasma exchange as a ‘hybrid’ therapy alongside other extracorporeal liver assist devices, before finally reviewing outstanding research questions for the future.

## Background

Paediatric acute liver failure (PALF) occurs when a patient with no prior liver disease demonstrates biochemical evidence of hepatic injury, accompanied by significant coagulopathy and/or encephalopathy [1]. Causes of ALF in children are varied; in a series of 215 consecutive admissions to a UK tertiary liver unit, around a third of cases were of indeterminate aetiology, with a quarter caused by drugs, and other common causes including viral hepatitis, Wilson’s disease, neonatal haemochromatosis, metabolic diseases, and autoimmune hepatitis [2]. This complicates the clinical course of children as varying aetiologies of PALF have different aetiology-specific management, with varying prognoses.

**Fig. 1:**
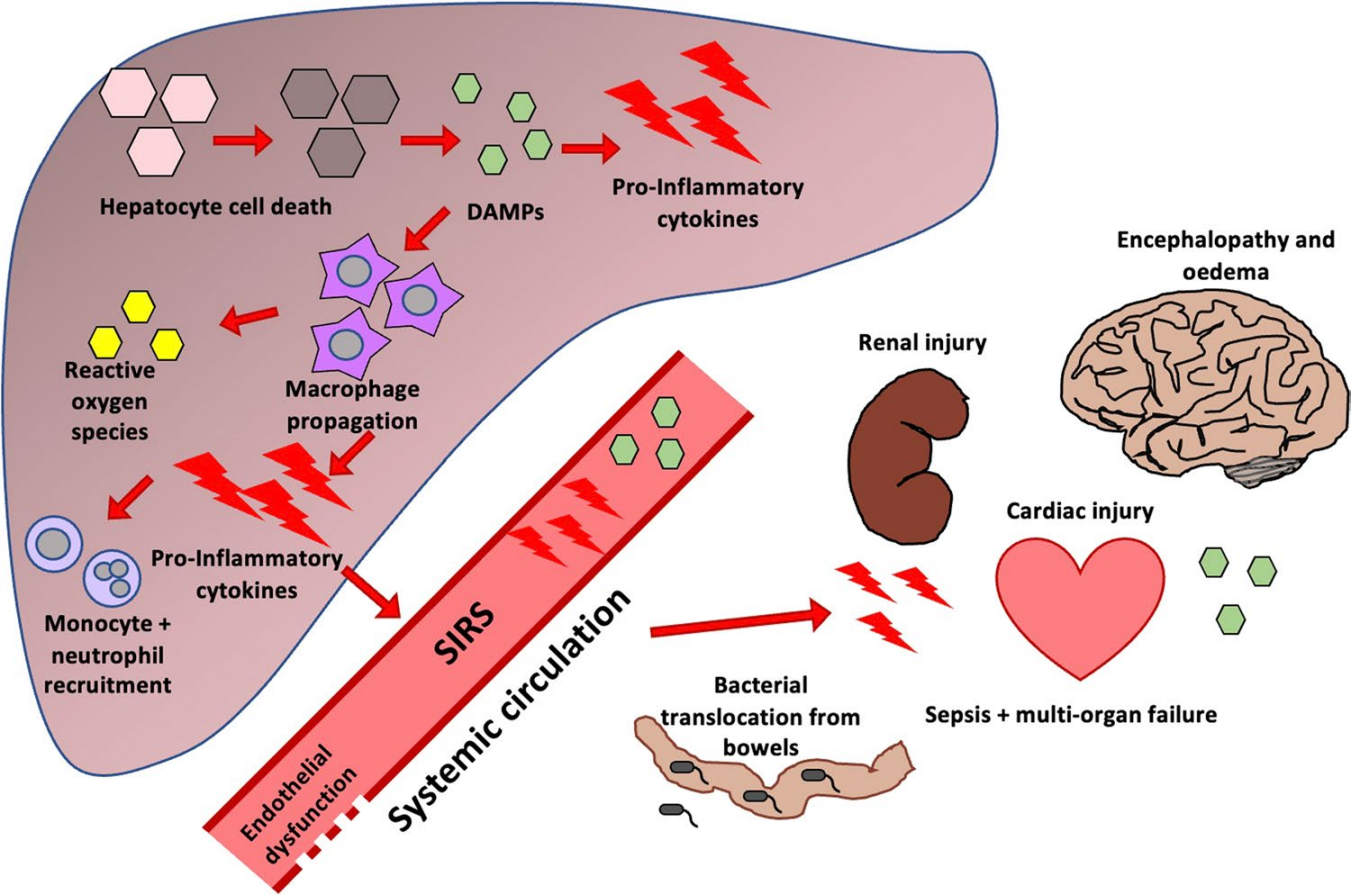
Propagation of early inflammatory response in ALF and spread of inflammatory mediators to the systemic circulation

Pathologically, PALF is characterised by hepatic necrosis, destruction of hepatocytes, and bile duct proliferation [3]. Hepatocyte damage leads to the release of damage-associated molecular pathogens (DAMPs), which provoke the activation of immune cells and an inflammatory cascade, with release of pro-inflammatory cytokines such as tumour necrosis factor (TNF)-α, interleukin (IL)-1β, and IL-6 [4]. Macrophages are influenced by the microenvironment and the presence of pro-inflammatory cytokines and toll-like receptor ligands in the initial hyperacute stage of PALF make macrophages pro-inflammatory, shifting their behaviour in a tissue-destructive fashion with further expression of pro-inflammatory cytokines [5] (see Fig. 1). In the later stages of injury, there is an increase in anti-inflammatory mediators, and macrophages shift function towards a pro-regenerative and pro-repair state. Neutrophils are also recruited to the damaged liver, but have impaired bactericidal function, making the patient more vulnerable to infections and sepsis; and this impaired function is correlated with non-survival without transplantation [6, 7].

Paediatric patients with ALF should receive early evaluation and transfer to a specialist liver unit, and those who develop significant encephalopathy or coagulopathy should be managed in intensive care [8]. Disease-specific therapies (such as N-acetylcysteine in paracetamol toxicity and antivirals in viral hepatitis) should be initiated as soon as possible. However, despite optimal management, morbidity and mortality remain high. In a 2008 study of 210 paediatric patients with ALF, 43% underwent liver transplant, 29% died, and the remaining 28% survived with their native liver (native liver survivors, NLS) [9]. Patients commonly experience significant complications including hyperdynamic circulatory failure, acute kidney injury, metabolic disturbances, coagulopathy, encephalopathy, and systemic inflammatory response syndrome (SIRS) driven by the release of cytokines and vasoactive mediators [5, 8] (Fig. 1). Multi-organ failure can result from this secondary immune response to damaged hepatocytes, as inflammatory mediators spread to the systemic circulation and toxic mediators are no longer cleared by the liver [6].

Prognosticating these children is challenging, in particular, distinguishing between those who will recover with medical therapy and those who require liver transplantation to survive. The King’s College Criteria (KCC), used in adults, does not reliably predict death in paediatric cohorts [10, 11]. Encouragingly, the trend over time suggests that an increasingly high percentage of patients are surviving with native liver with just medical therapy [12, 13]; however, the challenge is in identifying these children at an early stage and in the provision of bridging therapies. Bridging therapies give children time to allow the liver to spontaneously recover, and avert transplant, or to provide clinical stability and so bridge the patient until a liver becomes available. Useful bridging therapies could treat cerebral oedema by achieving clearance of ammonia [14] and reduce inflammatory mediators and SIRS – these being the complications most associated with mortality in ALF [15, 16]. There is increasing evidence suggesting that extracorporeal liver support (ECLS) systems can fill this role by supplementing the synthetic and detoxification roles of the liver and providing bridging either to liver recovery or to transplant.

## What are extracorporeal liver support systems?

ECLS systems support patients in ALF by purifying the blood of water-soluble and albumin-bound substances through a variety of methods, including albumin dialysis, plasma separation, plasma exchange, or a combination [17]. Meta-analyses from adult studies suggest that ECLS systems improve survival in ALF, with a calculated number needed to treat of eight to prevent one death [18].

Paediatric studies of ECLS systems tend to be small case series in critically ill paediatric patients admitted to ICU with ALF. However, some describe positive outcomes including bridging achieved to transplant or recovery. In 2008, six paediatric patients with ALF were treated with the molecular adsorbent recirculating system (MARS), a device combining albumin dialysis with haemodialysis, as a bridge to transplant; of these, two were bridged to transplant and survived, two survived without transplantation, and two died (one after transplantation) [19]. In a 2011 retrospective study of nine patients treated with single pass albumin haemofiltration, one patient was bridged until organ recovery, one avoided re-transplantation, six were bridged until liver transplantation, and overall three died (two post-transplant) [20]. Bilirubin and bile acid levels fell for all patients, and hepatic encephalopathy was generally reduced. Finally, a 2015 abstract described the use of Prometheus (a combination of high-flux haemodialysis with fractional plasma separation) in eight children with ALF, after which four patients were bridged to transplant, three were bridged to recovery, and an eighth patient died [21]. Considering the critically ill nature of these children, evidence of recovery or bridging to transplant with these devices, alongside the evidence of survival benefits in adults, is encouraging.

## What is plasma exchange?

Therapeutic plasma exchange (TPE) has been described as the form of artificial liver support that most closely mimics the function of the liver, by providing detoxification and synthetic function. It is one of the most well-studied ECLS modalities and, unlike other ECLS systems, has good evidence of efficacy in other immune-mediated conditions [22]. Typically, TPE involves the removal of 30–40 mL/kg of plasma (1–1.5 × plasma volume) from the patient via a double lumen central venous catheter, and exchange with isotonic human albumin solution (HAS), or fresh frozen plasma (FFP) before blood is returned to the venous circulation [23]. It can be performed via membrane filtration, whereby the plasma is separated from other blood components using hydrostatic forces against differently sized membrane filter pores, or by centrifugation, whereby plasma is separated from other blood components after rotation in a centrifugal bowl which sorts the different blood components into layers according to their density [24, 25]. High-volume plasma exchange (HVPE) is not consistently defined in the paediatric literature and is mostly expressed as an exchange of > 1.5–2.0 × estimated plasma volume [26, 27], but among adults, it has been defined as volume exchange equivalent to 10–15% of ideal body weight [28]. An exemplar plasma exchange regimen can be seen in Table 1.

**Table 1:** Regimen for plasma exchange in paediatric ALF

Setting	Access device	Removed from patient	Replacement fluid	Duration	Anticoagulation
Paediatric intensive care unit (PICU)	Double-lumen, central venous catheter. Rarely, large peripheral veins are used	Commonly 1–1.5 × plasma volume, whereby plasma volume = ((70–80 mL × (weight (kg))) × (1 – haematocrit)). High volume is often defined as exchange of 10–15% of ideal body weight, or > 1.5–2 × plasma volume	4.5% or 5% human albumin solution (HAS), or fresh frozen plasma (FFP), or a combination (higher fractions of FFP are given in ALF)	1.0 plasma volume in 1–2 h; 2.0 plasma volume in 4 h. Duration may vary depending on the patient, device, and complications	Unfractionated heparin (10–20 units per kg per hour) or prostacyclin (4–8 ng per kg per minute). In a bleeding child with ALF, the circuit can be run without any anticoagulation

## Complications

Complications of note from TPE include catheter-associated complications (haematoma, pneumothorax), hypotension, bacteraemia, thrombocytopenia, citrate accumulation, hypocalcaemia, haemolysis, inadvertent drug clearance, and anaphylaxis (to HAS or FFP) [29]. In a retrospective study of 48 children who received TPE in an Australian PICU 2007–2014, 21.2% of sessions involved a complication; the most common of these were circuit clotting (7.3%), access malfunction (4.0%), hypotension (3.8%), blood leakage (3.7%), and hypocalcaemia (0.8%) [30]. In total, 8.6% of patients experienced complications significant enough to necessitate discontinuation of TPE. Hypocalcaemia and metabolic alkalosis are notable complications. Citrate is commonly used as an anticoagulant in blood bank products including FFP and citrate chelates ionised calcium, leading to hypocalcaemia; in addition, its metabolism is impaired in liver failure, so accumulation of citrate can cause metabolic alkalosis [31]. In a study of 51 paediatric patients with ALF/acute-on-chronic liver failure (ACLF) who received continuous kidney replacement therapy (KRT), being a recipient of TPE was independently associated with citrate accumulation in a multivariate analysis [31].

## Mechanism of action in ALF

The removal of the patient’s plasma during plasma exchange leads to removal of inflammatory mediators including DAMPs and the cytokines, toxins, and metabolites that accumulate in the plasma secondary to impaired secretory and metabolic function of the liver. Large pore sizes allow for the removal of large molecules including immunoglobulins. The replacement of plasma with FFP allows the replacement of clotting factors and hence mimics the synthetic function of the liver. As described, this mechanism has led to TPE being employed routinely in immune-mediated disorders such as Guillain–Barré syndrome, Goodpasture’s syndrome, and thrombotic thrombocytopenic purpura [22]. Interestingly, a 2014 meta-analysis of two randomised controlled trials (RCTs) found that the use of TPE in adult patients with sepsis was associated with a significant reduction in all-cause mortality, although in the overall analysis including paediatric studies there was no significant difference in the relative risk of mortality [32]. Overall, knowledge of its mechanism of action, and evidence from other conditions, suggests that TPE could be well-placed to ameliorate the immune-mediated sequelae of PALF that result in increased likelihood of sepsis and SIRS. The Surviving Sepsis Campaign (SSC) guidelines (2020) describe in detail the immunologic basis of the use of TPE in children with septic shock associated with TAMOF (thrombocytopenia associated with multi-organ failure), though based on evidence, the panel could not recommend for or against the use of TPE in children with septic shock associated with TAMOF [33].

## High-volume plasma exchange

Damage to hepatocytes during ALF leads to a significant pro-inflammatory response, as described above, especially in the immediate phase after injury. A recent RCT in adults has shown that high-volume plasma exchange (HVPE) may be particularly effective in attenuating the inflammatory response of ALF through the removal of cytokines and DAMPs. This 2016 paper by Larsen et al. described a prospective RCT in 182 adult patients with ALF who were assigned to either standard medical therapy (*n* = 90) or standard medical therapy in addition to HVPE (8–12 L, or 15% of ideal body weight, exchanged with an equivalent volume of FFP per day per procedure) for three consecutive days (*n* = 92) [28]. The authors evaluated the impact of HVPE on the presence of immune cells and leucocyte subsets and found that HVPE led to a significant reduction of pro-inflammatory markers. For example, production of TNF-α, IL-8, and histone-associated DNA were significantly reduced in patients who received HVPE compared to those who did not, as were modulators of the inflammatory response including IL-4, IL-10, and TGF-β. IL-6 was reduced in those who received HVPE within 48 h of admission. The authors proposed that HVPE suppresses the innate immune response by reducing the levels of DAMPs, and thus the levels of inflammation induced by the innate immune response, and so reduces levels of cell and tissue death. This conclusion was corroborated by an observed reduction in SIRS score and sequential organ failure assessment score (SOFA) in the HVPE group compared to controls and significant differences in clinical outcomes. Most significantly, survival to hospital discharge was 58.7% in the HVPE group versus 47.8% in the control group (hazard ratio (HR) 0.56, 95% confidence interval (CI) 0.36–0.86, *p* = 0.0083). In addition, patients who had a poor prognosis but were not eligible for transplant due to contraindications had a significant increase in survival after the use of HVPE. On the basis of the clinical outcomes of this study, in 2017, the European Association for the Study of the Liver (EASL) incorporated plasma exchange into its clinical practice guidelines for the management of ALF, recommending early treatment to improve transplant-free survival, in the context of RCTs [34]. Subsequently, in 2019, the American Society of Apheresis recommended HVPE as a first-line therapy in ALF [29]; it also specifically recommends TPE in cases of fulminant Wilson’s disease.

## Practicalities and controversies

**Fig. 2:**
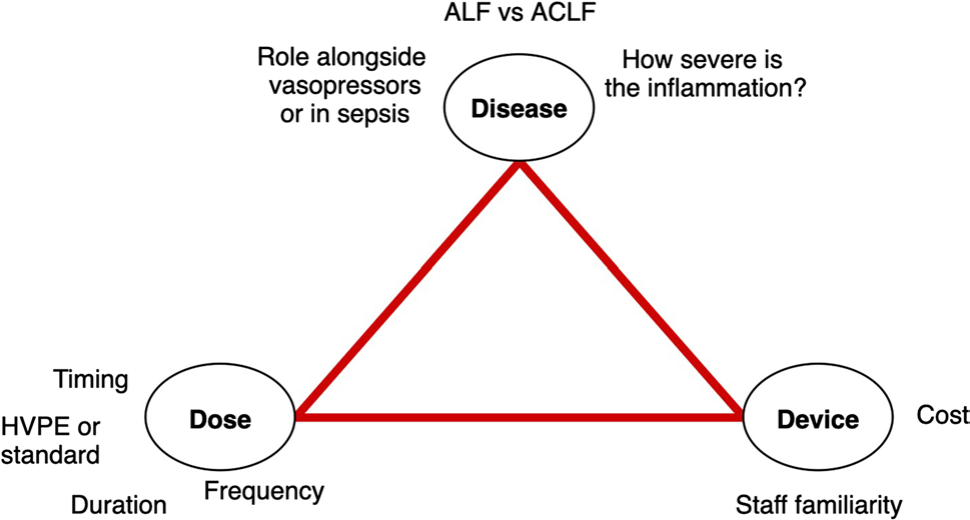
Three domains for consideration regarding TPE in ALF: what disease, what dose, and what device

The Larsen et al. study has provoked as many questions as it has answered regarding the role of TPE in ALF. First, how can we define the ‘dose’ of plasma exchange that is most beneficial? Despite the encouraging greater survival in the HVPE group, it is as-yet unknown whether HVPE confers improved outcomes over standard volume PE (SVPE) by preventing the side effects associated with large volumes of FFP. Similarly, the authors used a HVPE regimen for three consecutive days; could a longer or shorter regimen have given similar results? Secondly, there was no standardisation of the timing of initiation of the TPE sessions – some patients received HVPE on the day of ICU admission, whereas others received it later, which may have influenced outcomes. The timing of TPE (early vs. late) also determines what molecules in the disease process are being targeted – the significance of this is discussed in the next section. The next question: what is the ideal disease process to target that may benefit from TPE? In Larsen et al., 56% of the study population had paracetamol-induced ALF (similar in both groups), and extrapolation to rarer causes of ALF (viral, drug-induced, indeterminate, metabolic, etc.) may require further study [28]. Although this review focuses on ALF, it should be noted that trials are underway regarding the role of TPE in acute-on-chronic liver failure (e.g., the APACHE trial [35]). The next point for discussion is the ideal device for delivery of TPE. As described above, TPE can be administered either via membrane or centrifugal separation of plasma; there are no trials demonstrating superiority of centrifugal over membrane TPE in ALF, although membrane TPE may be more practical as it can be administered using devices that are present in ICU and which can also deliver KRT if required [36]. Lastly, Larsen et al. used FFP as the sole replacement fluid, but potentially supplementation with a proportion of human albumin could be beneficial as it has been shown that circulating albumin in liver failure could be dysfunctional [37]. The disadvantages of the HVPE approach using FFP include higher costs secondary to higher volumes of FFP and increased burden on the blood bank; a greater possibility of citrate toxicity and associated hypocalcaemia, hypomagnesaemia, and metabolic alkalosis [38]; and a greater likelihood of clearance of circulating drugs due to the higher volumes exchanged. The key outstanding questions regarding the role of TPE in liver failure are summarised in Fig. 2.

Maiwall et al. (2021) addressed some of the outstanding questions regarding the role of HVPE from the Larsen et al. study in an RCT comparing standard medical treatment against standard medical treatment plus standard volume plasma exchange (1.5–2.0 × plasma volume), in a cohort of 40 patients with non-paracetamol-induced ALF and cerebral oedema [39]. Rather than running a fixed duration of three consecutive days of PE as in the Larsen et al. study, the number of sessions was determined in each individual patient according to their clinical response. The study found that standard volume TPE was safe and effective and was again associated with a significantly higher 21-day transplant-free survival compared to standard medical treatment (75% vs. 45%, HR 0.30, 95% CI 0.01–0.88, *p* = 0.04). The authors again demonstrated that the use of TPE was associated with a decrease in pro-inflammatory cytokines and reported only mild adverse effects. Considering the costs and other theoretical risks associated with HVPE, this study is of interest and highlights the value of standard volume plasma exchange in and of itself. Ideally, a future three-armed RCT would compare both high- and standard volume modalities against standard medical treatment.

## Timing of TPE

**Fig. 3:**
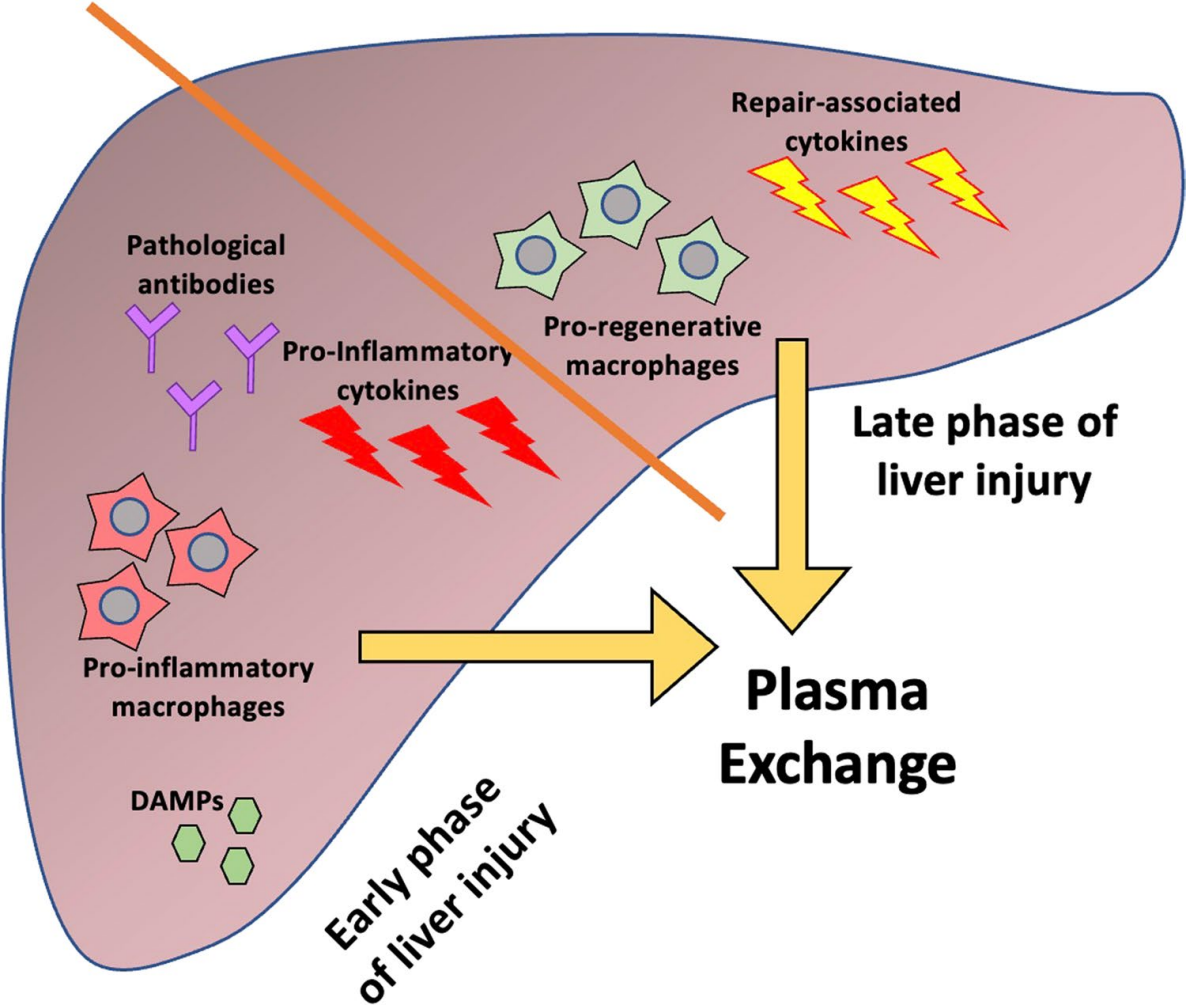
Relative phase of inflammatory response in the liver according to timing of liver injury and the consequent action of plasma exchange

As the molecules involved in the massive inflammatory response in the initial phase of acute liver injury are the same as those which initiate the regenerative process, a significant question is whether the mechanism of TPE and HVPE could potentially be disadvantageous for certain subgroups of patients, by removing molecules that are actively involved in regeneration, and whether modifying the timing of HVPE could mitigate this risk (see Fig. 3). This concern was probably part of the reason why Larsen et al. planned to administer HVPE for 3 days only (and no longer) as part of their trial [28].

The EASL recommendation of *early* treatment with HVPE in ALF was informed by the results from Larsen et al. showing particular improvement in parameters following early HVPE (initiated < 48 h following ICU admission) [28, 34]. As described above, in the hyperacute stage of ALF, macrophages are polarised in a pro-inflammatory state which leads to tissue damage, and spill-over of cytokines and vasoactive mediators at this stage leads to SIRS and multi-organ failure. Removing inflammatory molecules at this early stage, therefore, may well be most beneficial, rather than at a later stage where macrophages are primed to promote liver regeneration [5]. This idea leads to the intriguing possibility of using biomarkers which can define the stage of hepatic injury, the timing of extracorporeal therapies, and the prognosis. This idea has been best studied in paracetamol-induced liver injury, and serum neopterin and soluble CD163 have been suggested as markers of the maximal phase of macrophage activation in ALF [40]. However, further research will be required as to whether using biomarkers to guide treatment will be useful in routine clinical practice, either for adults or for children.

## Indications for TPE in paediatrics

In adults, the selection of patients for TPE is somewhat dependent on institutional practice. In our centre, TPE is primarily used in adult ALF patients fulfilling listing criteria who are not fit for transplant or with contraindications to listing, and hepatic encephalopathy must be present, with TPE initiated as soon as possible after the development of hepatic encephalopathy.

The evidence base for the use of TPE in PALF is less well-established, and at present, it is mostly employed on a case-by-case basis. Based on its mechanism of action, TPE has been used in cases of medically refractory coagulopathy; to avoid fluid overload from blood products; in children with PALF in multi-organ failure as an immunomodulatory therapy; to provide bridging to patients awaiting liver transplant, or for bridging towards spontaneous regeneration of the liver by dampening SIRS; and in patients with immune-mediated ALF. In some centres, TPE is used in Wilson’s disease. In the following section, we will discuss the key studies of TPE in paediatric patients.

## Evidence for plasma exchange in paediatrics

Relevant to paediatric intensivists and hepatologists, there is an increasing amount of evidence regarding the safety and feasibility of HPVE in PALF, largely from retrospective observational case series or cohort studies. The varied focuses of these studies mean it is difficult to delineate an ideal target population for clinical practice; in children, the causes of ALF are more varied than in adults. As described above, it is unclear whether the ideal patient population to target would include those with immune-mediated ALF, Wilson’s disease, or sick children who are on multiple inotropes and vasopressors, suggesting significant SIRS as a component of their illness. Studies also undertake exchange of variable plasma volumes. In the following section, we review the studies describing TPE in PALF.

Most studies in paediatrics have described the use of higher-volume exchanges in ALF, but in 2016, a retrospective case series, Pham et al., described the use of standard volume TPE (exchange of 1–1.25 × plasma volume in the majority of sessions) in ten patients who developed ALF secondary to Wilson’s disease [41]. The patients were part of the American Society for Apheresis Wilson’s Disease Apheresis Registry. Replacement fluid was FFP for 77% of exchanges (33/43), and the remaining 23% used a combination of plasma with 5% albumin. Five patients had mental status changes at the time of TPE. Secondary to TPE, 10% of exchanges were associated with adverse events (citrate toxicity, and a febrile reaction in one case). At 6 months of follow-up, all ten patients had survived, with nine out of 10 (90%) having undergone liver transplantation. This is an encouraging outcome – but notably there was no control group, and the procedures implemented for each patient varied. Considering Wilson’s disease, an added benefit of TPE is that it also removes copper, at an average of 20 mg per session [29]. Among other studies of paediatric patients with Wilson’s disease, a 2010 abstract of 37 TPE sessions for 14 children, four of whom had Wilson’s disease, described TPE (volume exchanged not stated) resulting in significant improvements in biochemical parameters including total bilirubin, INR, AST, ALT, and ammonia, without significant adverse events [42].

Among papers demonstrating high-volume exchange in paediatrics, in 2001, Singer et al. performed a retrospective review of 49 paediatric patients with ALF or ACLF aged between 10 days and 18.4 years who underwent 243 episodes of plasma exchange, with removal of mean 2.2 + / − 0.6 times plasma volume, with FFP as the replacement solution [43]. The patients received daily TPE until they either recovered, died, or were transplanted. They experienced significant improvement in coagulation parameters, including increases in fibrinogen and factors II, V, VII, and IX; the prothrombin times of patients who received daily TPE did not exceed 25 s. Total bilirubin and transaminases also fell post-TPE, but neurologic examination results were not significantly altered. Of clinical outcomes, 17 (35%) were not transplanted, of whom only 3 (6%) recovered and achieved NLS, and the remainder died; 32 (65%) were transplanted, of whom 17 (35%) were alive at follow-up. The retrospective nature of the study, the inclusion of patients with ACLF, and the high proportion of patients included who received TPE prior to 1995 (when LT was prioritised at the centre) limit conclusions that can be drawn. However, encouragingly, there were again only minimal complications; none of the 243 sessions was associated with significant internal or catheter site bleeding, hemodynamic instability, or systematic organ failure.

A retrospective cohort study was published in 2019 of 23 patients aged under 18 with ALF, of whom 18 received TPE [44]. The patients received TPE using FFP at 2–4 times their estimated plasma volume, for three successive days, and subsequently once every 2 or 3 days depending on their clinical state. Overall, 11 (48%) of the cohort attained NLS, nine (39.1%) died without transplant, and three were transplanted (of whom one died later). The authors observed that the NLS group had fewer sessions of TPE than the non-NLS group (3 vs. 9, *p* < 0.01) and that a cut-off criterion of ≤ 6 TPE sessions had a sensitivity of 100% and specificity of 66.7% for NLS. However, the retrospective case series design, and consequent confounding by indication, limits the conclusions that can be drawn from this study. In addition, it was not reported how many days after admission that TPE was initiated, and therefore whether TPE was initiated during the hyperacute phase of these patients’ illness, when it may have been more beneficial, or after.

Two further studies have been published in the past year evaluating plasma exchange in a paediatric cohort. The first, Pawaria et al., was a 2021 prospective non-randomised study in patients under 18 with ALF secondary to Wilson’s disease [26]. The study compared 19 patients who received plasma exchange of > 1.5 × plasma volume (defined by the authors as HVPE) for a minimum of three consecutive days to 18 patients who received standard medical treatment. Patients who consented to receive plasma exchange alongside standard medical treatment were enrolled in the plasma exchange group, and those who refused consent were given standard medical treatment alone. The primary outcome was transplant-free survival at 90 days after enrolment. Overall, 47.3% of the plasma exchange group had transplant-free survival compared to 16.7% of the standard medical treatment group (Odds Ratio (OR) 2.84, 95% CI 0.91–8.8, *p* = 0.049). Among study limitations, the study was non-randomised, and the patients included those with ACLF as well as ALF. In addition, patients with sepsis were excluded, even though outcomes in this group may have been valuable considering the immune-mediated pathology of ALF. However, despite these limitations, it is notable that this study showed a significantly greater odds ratio of survival after plasma exchange in PALF.

The second 2021 study of HVPE in PALF aimed to establish the safety and feasibility of HVPE in this cohort [27]. The authors described a retrospective analysis of 16 children with ALF who each received at least one series of three treatment sessions with HVPE, whereby 10% of body weight was exchanged with FFP for three consecutive days. Children were referred for HVPE either due to bilirubin levels exceeding 200 umol/L or toxic aetiology for their ALF or both. Complications were minimal, with no bleeding-related complications, and no electrolyte or acid–base disturbances other than three children who developed alkalosis (pH > 7.55) that responded to treatment. Bilirubin, ALT, and INR all underwent significant declines during HVPE treatment. Eight of 16 children avoided transplantation, two survived after receiving a transplant, and six died (at a range of 8 to 254 days after the final HVPE session). The non-randomised observational nature of the study precludes any conclusions regarding whether HVPE altered outcomes for these children, but the safety and feasibility data are again encouraging. We would recommend future studies to document how many days after ALF onset that standard volume PE or HVPE is implemented, which would help contextualise results within our current understanding of the different stages of the inflammatory process in ALF. A summary of the evidence for plasma exchange in paediatric ALF can be seen in Table 2, including all studies known to ourselves with a minimum of ten patients**.**

**Table 2:** Summary of key studies describing plasma exchange in paediatric ALF

Citation	Time-frame	Country	Study design	Population	Details of TPE modality	Mortality and liver survival	Other key outcomes
Chien et al. 2019 [44]	2003–2016	Taiwan	Retrospective observational cohort study	*n* = 23 (total ALF), *n* = 18 (received TPE)	Exchange volume of 2–4 times estimated plasma volume; daily for three days then variable according to clinical condition	11/23 (48%) had native liver survival 9/23 (39%) died without transplant 1/23 (4%) died post-transplant 2/23 (9%) survived post-transplant	The NLS group had fewer sessions of TPE than the non-NLS group (3 vs. 9, *p* < 0.01)
Demirkol et al. 2010 [42]	2005–2009	Turkey	Retrospective observational case series	*n* = 14 (ALF)	Not described in detail (abstract). Fourteen patients underwent a total of 37 TPE sessions	4/14 (29%) had native liver survival 5/14 died (36%) 5/14 (36%) had liver transplants	Biochemical variables improved when comparing pre-TPE and post-TPE values; no patients experienced serious adverse events
Jørgensen et al. 2021 [27]	2012–2019	Denmark	Retrospective observational cohort study	*n* = 16 (ALF)	Fluid volume corresponding to 10% of body weight was exchanged with FFP; sessions for 3 consecutive days followed by re-assessment	8/16 (50%) had native liver survival 5/16 (31%) died without transplant 1/16 (6%) died post-transplant 2/16 (13%) survived post-transplant	There were no bleeding-related complications, and no electrolyte or acid–base disturbances other than three children who developed alkalosis. Bilirubin, ALT and INR significantly declined with HVPE treatment
Pawaria et al. 2021 [26]	2014–2019	India	Prospective nonrandomised interventional study	*n* = 37 (ALF due to Wilson’s) in total; n = 19 received HVPE, *n* = 18 received standard treatment	Exchange in one session of > 1.5 × plasma volume; exchange for three consecutive days (maximum 3–6 sessions)	4/19 (21%) of HVPE group and 5/18 (28%) from standard treatment group were transplanted 9/19 (47%) of HVPE group and 3/18 (17%) of standard treatment group had transplant-free survival	47.3% of the HVPE group had transplant free survival compared to 16.7% of the standard medical treatment group (OR 2.84, 95% CI 0.91–8.8, *p* = 0.049)
Pham et al. 2016 [41]	2000–2014	USA	Retrospective observational case series	*n* = 10 (ALF due to Wilson’s)	Plasma exchange of 1–1.25 plasma volume for 42 of 43 exchanges; each patient had a median of 3.5 procedures (range 1–9)	1/10 (10%) had native liver survival 9/10 (90%) survived post-transplant (follow-up of 6 months)	Of 43 TPE procedures, 70% required calcium supplementation, and 10% reported adverse events
Singer et al. 2001 [43]	1987–2000	USA	Retrospective observational case series	*n* = 49 (ALF or ACLF)	Plasma volume of 2.2 + / − 0.6 removed, replaced with 74 + / − 11% FFP; daily until recovered, died or transplanted	3/49 (6%) had native liver survival 14/49 (28%) died without transplant 15/49 (31%) died post-transplant 17/49 (35%) survived post-transplant	No change in neurological examination results. Significant improvement in coagulation, total bilirubin, and transaminases post-TPE. No major complications
Hybrid/combination approaches							
Akcan Arikan et al. 2018 [45]	NS (24 months)	USA	Retrospective observational cohort study	*n* = 15 (ALF or ACLF)	Continuous veno-venous haemodiafiltration, centrifugal plasma exchange with FFP at 1.3–1.5 × plasma volume, and MARS	2/15 (13%) had native liver survival 4/15 (27%) died without transplant 9/15 (60%) survived post-transplant	13/15 (87%) of patients had improved hepatic encephalopathy grade, including all survivors
Ide et al. 2015 [46]	2006–2011	Japan	Retrospective observational cohort study	n = 17 (ALF)	Continuous veno-venous haemodiafiltration, plasma exchange and liver transplantation. Plasma exchange used 100 mL/kg of FFP once daily until coagulopathy recovered	15/17 (88%) survived post-transplant 2/17 (12%) died post-transplant	11/15 survivors (73%) had no neurological morbidities
Rodriguez et al. 2017 [31]	NS (30 months)	USA	Retrospective observational cohort study	*n* = 51 (ALF or ACLF, total); *n* = 20 received plasma exchange	Continuous veno-venous haemodiafiltration; 20 patients also received 5.8 + / − 3.8 plasma exchange sessions, with FFP placement. Plasma exchange was centrifugal and 1–1.5 × plasma volume	Of whole cohort, 29/51 (57%) died in hospital 26/51 (51%) received liver transplants	Patients receiving plasma exchange were more likely than non-recipients to have citrate accumulation than non-plasma exchange patients (*p* = 0.004)
Schaefer et al. 2011 [47]	2002–2010	Germany	Retrospective observational cohort study	*n* = 10 (ALF or ACLF)	MARS (standard for *n* = 7, Mini for *n* = 3). This was alternated with combined plasma exchange and haemodialysis (for *n* = 8). Plasma exchange was 1.5 × plasma volume	5/10 (50%) died 3/10 (30%) others were successfully transplanted 2/10 (20%) had native liver survival	Patients showed significantly greater reductions in bilirubin, ammonia and INR on PE/HD than on MARS/mini-MARS (*p* < 0.05)
Tufan Pekkucuksen et al. 2020 [48]	2013–2016	USA	Retrospective observational cohort study	*n* = 63 (*n* = 20, 32% TPE for ALF or ACLF)	Tandem continuous veno-venous haemodiafiltration and plasma exchange. For plasma exchange; mean exchange volume 1.34 + / − 0.21. FFP used for liver failure patients	25/63 (39.7%) died, transplant outcome not stated	Non-survivors had significantly greater time to initiation of TPE from PICU admission, than survivors (*p* = 0.029)

*ACLF* acute-on-chronic liver failure, *ALF* acute liver failure, *ALT* alanine aminotransferase, *AST* aspartate aminotransferase, *FFP* fresh frozen plasma, *HVPE* high-volume plasma exchange, *ICU* intensive care unit, *INR* international normalised ratio, *KRT* kidney replacement therapy, *MARS* molecular adsorbent recirculating system, *NLS* native liver survivors, *NS* not stated, *PE/HD* plasma exchange/haemodialysis, *PICU* paediatric intensive care unit, *PT* prothrombin time, *TPE* therapeutic plasma exchange

## Hybrid approaches in paediatrics

Several paediatric studies have investigated hybrid approaches, most commonly the combination of plasma exchange with continuous KRT, in combined cohorts of patients with ALF and ACLF. Hybrid approaches allow for the removal of differently sized molecules; for example, the combination of continuous KRT with plasma exchange allows for the removal of small water-soluble molecules such as ammonia, in addition to toxic molecules with large molecular mass which are removed using TPE [49].

Among key studies of hybrid approaches in paediatrics, in 2011, a retrospective observational study was published analysing ten children (0.1–18 years) with ALF/ACLF, comparing their responses to MARS or MARS Mini, with their responses to treatment with combined TPE and haemodialysis (PE/HD) [47]. The authors found that the use of PE/HD had superior efficacy versus MARS/MARS Mini, with greater biochemical improvement in total and unconjugated bilirubin, INR, and ammonia (*p* < 0.05). A 2015 study of infants under 12 months who developed ALF (*n* = 17), described a combination approach of continuous veno-venous haemodiafiltration (CVVHDF) until liver transplantation, alongside plasma exchange of 100 mL/kg of FFP once a day for 6–8 h until recovery of coagulopathy [46]. Overall, 15 of 17 infants survived at a median follow-up of 28 months, all survived to discharge from ICU, and 11 of 15 survivors experienced no neurological morbidities. Positive neurological outcomes were also described in a 2018 study of 15 paediatric patients who received a combination of CVVHDF, standard volume plasma exchange, and MARS, wherein 13 of 15 patients (including all 11 survivors) had improvement in their grade of hepatic encephalopathy after treatment [45]. Finally, in 2020, an observational study reported on 63 paediatric patients who received TPE and CVVHDF, of whom 33% received TPE to treat complications of ALF/ACLF [48]. The study found that time to initiation of TPE was longer in non-survivors (who had time to initiation of 4 days (2, 13), compared to 2 days (1, 3) in survivors, *p* = 0.029). This finding is of interest considering the evidence previously discussed regarding the role of TPE in removing pro-inflammatory mediators in the early stage of ALF and suggests that, for children as well as for adults, early treatment with TPE is more likely to be beneficial.

**Fig. 4:**
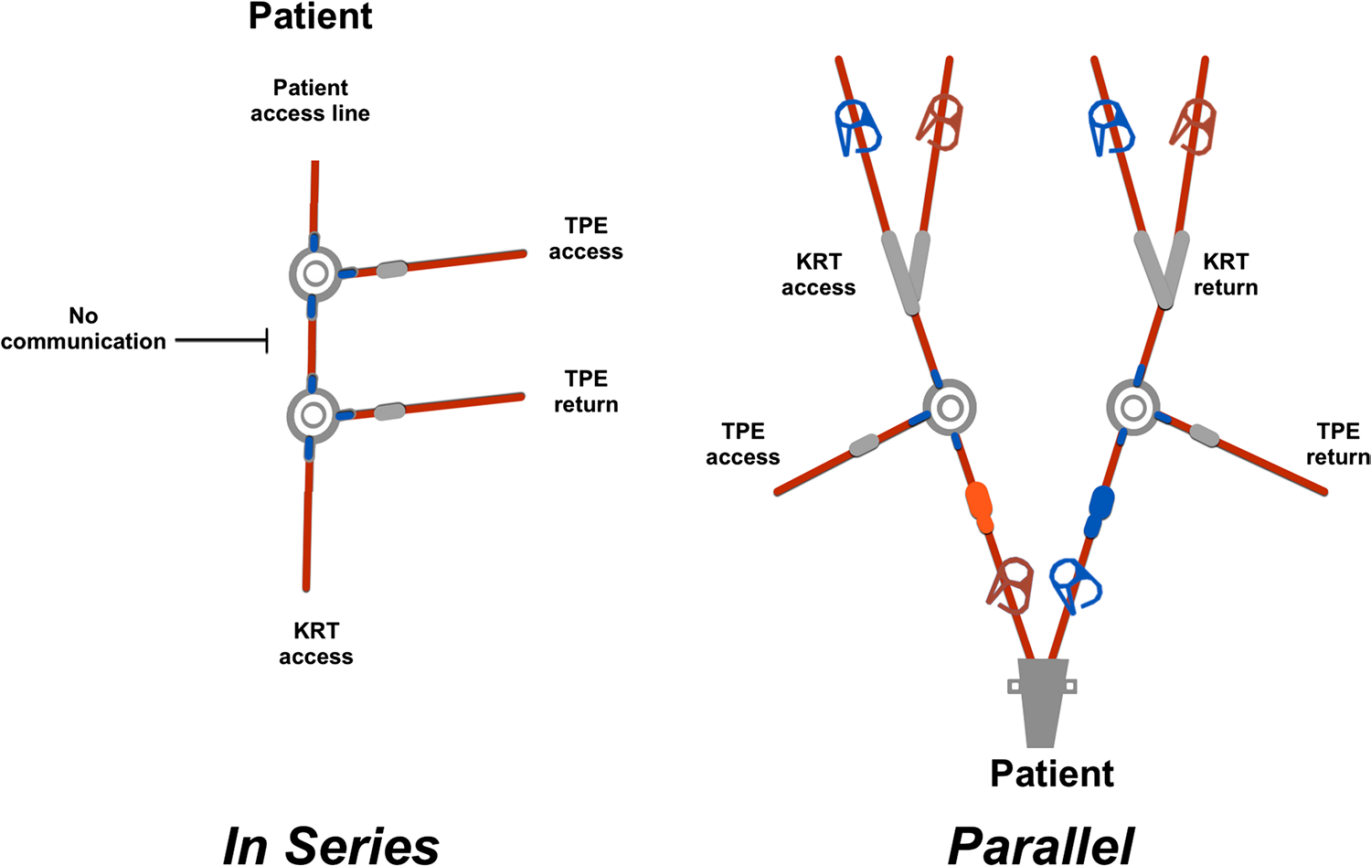
Illustration of using one vascular access for TPE and KRT in series (left, using the access lumen) and in parallel (right, on separate catheter lumens)

Despite these outcomes, it is important to be mindful of the cost and technical expertise required to utilise multiple ECLS systems together. There are also practical difficulties arising from hybrid therapies. Simultaneous plasma exchange and continuous KRT in adults are achieved usually with two vascular access devices in two different sites, which are much more achievable in adults than in children, where vascular access is much more difficult. The same vascular access can be used if continuous KRT is paused for the duration of the TPE, but risks rebound of ammonia. Simultaneous TPE and continuous KRT can be performed using the same vascular access either in parallel, when the TPE access and return lines are on separate lumens of the vascular catheter, or in series, when both TPE access and return lines are on the same lumen (the access lumen) of the catheter (Fig. 4). Addressing practicalities to optimise treatments which can be offered to children with ALF will be important to improve outcomes in future.

## Conclusions

In conclusion, therapeutic plasma exchange has potential as an important bridging option in early paediatric ALF, either as a bridge to liver transplant or as a bridge to spontaneous recovery with native liver. It has value through its immunomodulatory effect in ALF where inflammation plays an important role. Patients will be aided by future research that addresses the following questions – what are the indications in PALF to initiate TPE especially with varying aetiologies? What is the optimal timing of plasma exchange to best complement the body’s immune response? Can biomarkers guide timing of plasma exchange by predicting illness stage and trajectory (biomarkers of cell death-necrosis and apoptosis for the early hyperacute inflammatory stage, and biomarkers of regeneration like alpha fetoprotein, phosphate, and anti-inflammatory cytokines for the resolution phase)? Finally, should TPE be incorporated as the standard of care in combination with continuous KRT in particular subsets of children? There are several unexplored questions regarding the use of TPE in PALF which require further research via multi-centre collaborations to guide optimal timing and treatment for young patients presenting with ALF.

### Key summary points

1. Paediatric ALF is a rare condition with a high mortality; it is increasingly recognised that immune system deregulation contributes to the significant complications of ALF (sepsis, multi-organ failure, cerebral oedema).
2. In view of the limited supply of livers, and the regenerative capacity of the liver, increasingly, attention has been given to ‘bridging’ therapies which can support the patient until they regain function of the liver or to transplant.
3. Plasma exchange is the ideal extracorporeal liver support system, by providing detoxification and synthetic function.
4. Recent randomised controlled trials in adults have demonstrated increased survival among patients receiving plasma exchange and illustrated that this has occurred alongside reduction in pro-inflammatory markers.
5. The evidence for plasma exchange in paediatrics, primarily from case series and cohort studies, shows that this therapy has potential to improve morbidity and mortality for critically ill children with ALF in PICU; more research is needed to confirm this theory.

## Multiple choice questions (answers given following the references)

1. Which technique(s) is most commonly used to perform plasma exchange?
  - Centrifugation
  - Membrane filtration
  - Centrifugation or membrane filtration
  - Sedimentation
  - Precipitation
2. In which specific condition causing ALF does the American Society of Apheresis recommend TPE as a first-line therapy?
  - Paracetamol-induced liver injury
  - Wilson’s disease
  - Viral hepatitis
  - Autoimmune hepatitis
  - Metabolic disorders
3. Key potential complications of plasma exchange include:
  - Drug accumulation
  - Thrombocytosis
  - Polycythaemia
  - Leukopaenia
  - Hypocalcaemia
4. Which is an accurate equation for plasma volume?
  - (70–80 mL×(weight (kg)))×(1 – Haematocrit)
  - 70 mL×(weight (kg))
  - (80 mL×(weight (kg)))×(Haematocrit)
  - 100 mL×(weight (kg))
  - Hb/100×(80 mL×(weight (kg)))
5. Which of these is a potential patient subgroup for TPE in children with ALF?
  - Immune-mediated ALF
  - Wilson’s disease
  - Refractory coagulopathy
  - Child with SIRS
  - All of the above

## Data Availability

Data sharing is not applicable to this article as no datasets were generated or analysed during the current paper.
